# Data Analysis and Forecasting of the COVID-19 Spread: A Comparison of Recurrent Neural Networks and Time Series Models

**DOI:** 10.1007/s12559-021-09885-y

**Published:** 2021-06-03

**Authors:** Daniela A. Gomez-Cravioto, Ramon E. Diaz-Ramos, Francisco J. Cantu-Ortiz, Hector G. Ceballos

**Affiliations:** School of Engineering and Sciences, Tecnologico de Monterrey, Monterrey, 64849 N.L. Mexico

**Keywords:** Covid19, Data science, Time series forecasting, Recurrent neural networks.

## Abstract

To understand and approach the spread of the SARS-CoV-2 epidemic, machine learning offers fundamental tools. This study presents the use of machine learning techniques for projecting COVID-19 infections and deaths in Mexico. The research has three main objectives: first, to identify which function adjusts the best to the infected population growth in Mexico; second, to determine the feature importance of climate and mobility; third, to compare the results of a traditional time series statistical model with a modern approach in machine learning. The motivation for this work is to support health care providers in their preparation and planning. The methods compared are linear, polynomial, and generalized logistic regression models to describe the growth of COVID-19 incidents in Mexico. Additionally, machine learning and time series techniques are used to identify feature importance and perform forecasting for daily cases and fatalities. The study uses the publicly available data sets from the John Hopkins University of Medicine in conjunction with the mobility rates obtained from Google’s Mobility Reports and climate variables acquired from the Weather Online API. The results suggest that the logistic growth model fits best the pandemic’s behavior, that there is enough correlation of climate and mobility variables with the disease numbers, and that the Long short-term memory network can be exploited for predicting daily cases. Given this, we propose a model to predict daily cases and fatalities for SARS-CoV-2 using time series data, mobility, and weather variables.

## Introduction

As referenced by the World Health Organization, the first case of COVID-19 was in Wuhan, China, on December 31, 2019 [1]. On May 21, 2020, there had been over 5,102,424 confirmed cases, which resulted in more than 332,924 fatalities around the world [2]. The pandemic is severe, and it continues to affect billions of people.

In this study, we compare three curve fitting models: linear, polynomial, and generalized logistic model (GLM) and two multivariate time series models: a long-short term memory (LSTM) neural network and a traditional time series, vector autoregression (VAR) model to explore the behavior of COVID-19 daily cases and fatalities in Mexico.

This study’s motivation is to contribute to the knowledge necessary to fight the disease and characterize its course in Mexico, with the attempt to display more preparedness and promote more logical actions by the policymakers and the population in general.

The generalized logistic model has been successfully applied in other studies to describe previous epidemics [3]. The LSTM algorithm, which uses a type of recurrent neural network (RNN), was previously used in other studies to predict infections over time [4]. Risk factors such as climate features and adherence to social distancing were previously hypothesized to affect the number of daily cases. However, we did not find a previous study analyzing the significance of these factors using machine learning techniques in time series forecasts.

For the data exploration and model training, we used the dataset obtained from the Resource Center at the John Hopkins University of Medicine GitHub repository [5]. We supplemented it with information about climate information obtained from the Weather Online API [6] and the social mobility rate obtained from Google’s COVID-19 Community Mobility Reports [7].

This paper’s remainder is structured as follows: Section 1 describes related studies on the topic, and Section 2 describes the methods and dataset used in the research. In Section 3, we present data exploration and preparation for modeling. Section 4 presents the results of the models. Section 5 presents the discussion of the products and future directions for this project. Lastly, Section 6 presents the conclusions of this study.

### Related Work

With the same purpose of forecasting COVID-19 confirmed cases, we were able to identify the following related work, which mainly consists of studies using multivariate time series regressions and curve-fitting models.

The related work includes the work of Chae, Kwon, and Lee [4] who compared a deep neural network and LSTM with the ordinary least squares methods (OLS) and the autoregressive integrated moving average (ARIMA) to predict three infectious diseases (chickenpox, scarlet fever, and malaria). This study showed that both deep learning models had better performance than the traditional OLS and ARIMA methods, with an average of 20% improvement on the root-mean-square error (RMSE).

A second related work is Liu et al. [8], who analyzed the impact of meteorological factors on COVID-19 in China’s provinces. The results obtained from this study indicated that the transmission could be affected by factors such as low temperature, low humidity, and mild diurnal temperature range. Thirdly, this study is related to the work of Chakraborty and Ghosh [9], who forecasted the number of COVID-19 cases for multiple countries, Canada, France, India, South Korea, and the UK. The research uses traditional time series models and analyzes the demographical features affecting the spread in these countries, showing how a conventional ARIMA model can describe the spread’s nonlinear and nonstationary behavior in various countries.

This work is similar to the work done by Tomar and Gupta [10] and the recent work performed by Chimmula and Zhang [11]. In the former study, curve-fitting methods and LSTM were used to predict the number of COVID-19 cases in India and measuring how preventive steps like social isolation and lockdown affected the spread of COVID-19. The results indicated that the preventive measures (social isolation and lockdown) worked well in containing the virus in India. The study also included a graph showing how the forecasted numbers with the logistic curve fitting closely resembled the official data. The latter paper applied LSTM networks to predict the termination point of the outbreak for Canada, achieve an RMSE of 45.7 for long-term predictions, and forecast that the potential ending point would be around June 2020.

Regarding statistical models, Schuttler et al. [12] analyzed the spread of COVID using a sigmoid function. The authors showed how this simple fitting model could help estimate the diseases’ peak in many European countries and China. These results were similar to the ones in Andreas et al. [13], which determined Italy’s cases’ inflection point and obtained a coefficient of determination of 0.99. These studies on the COVID pandemic show how the disease’s growth can be described with an S-shaped curve and how the data can be fitted by applying traditional nonlinear least squares to the equation [14]. Furthermore, there have been previous studies where this function has been used in predicting other epidemic diseases [15, 16].

Moreover, previous studies [17–19] on the application of the susceptible–exposed–infected–removed (SEIR) framework (or some variations) on the data of COVID-19 confirmed cases solely for its prediction. Undoubtedly, the analysis of this literature has covered a wide range of topics on applying epidemiological methods. Nevertheless, these models are subject to limitations, such as not providing the impact and interaction of additional variables [20]. This has provided a potential area of opportunity for the data-driven techniques of machine learning. The present study contributes to the literature by applying machine learning techniques to identify the pandemic impacts and compare these techniques with the more traditional time series models.

## Methods

This research compares different techniques to forecast COVID-19 incidences and obtain insights into the COVID-19 outbreak. The exploration and visualization of the data and the machine learning modeling were performed using Python programming and ran in the open-source Jupyter Notebook platform. The program is available on GitHub^1^.

### Datasets

The dataset used for this analysis comes from the Resource Center at the John Hopkins University of Medicine. The data collected is open source and is available through a GitHub Repository [5], which is updated daily at 9 am EST.

The dataset contains information for the accumulated confirmed cases and fatalities in 173 countries. The following features are available in this data set:

- Country: Provided for 173 countries,
- Province: Only for Australia, Canada, China, Denmark, France, Netherlands, United Kingdom, United States,
- Date: Days since January 22, 2020, to March 31, 2020 (70 days),
- Confirmed Cases: Total number of confirmed COVID-19 cases, and
- Fatalities: Total number of deaths.

The John Hopkins data were supplemented with additional covariates: climate variables and social mobility rates. The weather information was extracted from the Weather Online API [6], and the variables include max temperature, min temperature, UV index, humidity, precipitation, pressure, and wind speed. In contrast, the social mobility rate was obtained from Google’s COVID-19 Community Mobility Reports [7]. It includes the following variables, which represent a percent change from the baseline: retail and recreation, grocery and pharmacy, parks, transit stations, workplaces, and residential. These rates show how visits and stays differ from the baseline, which is the week’s corresponding day’s median value.

### Univariate Growth Curve Models

The growth curve models, also called curve fitting models, are multilevel models mainly used to describe how a continuous outcome changes over time, focused on the between-individual variations [21]. Different types of these models include linear, polynomial of various degrees, logarithmic curve fit, and nonlinear curve fit  [22].

In this paper, we used three different growth models: linear regression, polynomial regression, and generalized logistic regression. We fitted them the data of the confirmed accumulated cases and fatalities in Mexico to identify the mathematical function that provided the best fit to the line or curves in the dataset. The linear regression approximates a straight line, while polynomial and generalized logistic regression are nonlinear regressions that approach the data by a curved equation.

The curve fitting models hypothesize that the GLM adjusts better to the population growth (COVID-19 cases). By obtaining the lower part of the curve, we can acquire the function parameters and get the complete curve, which can help us estimate the inflection point and limiting value.

The equations for each of these techniques are listed below. Equation (1) is for linear regression and considers the slope of the line as *a* and *b* as the intercept of the value of *f*(*x*) when $x = 0$. Equation 2 shows the fitting of a polynomial regression with *a* being the set of coefficients and *n*, the polynomial degree. Finally, Equation 3 considers *e* as Euler’s number, $x_0$ as the x value of the sigmoid’s midpoint, *L* as the curve’s maximum value, and *k* is the logistic growth rate.

1 $$\begin{aligned} f(x) = ax + b \end{aligned}$$

2 $$\begin{aligned} f(x) = \sum _{n=0}^{i} a_nx^n \end{aligned}$$

3 $$\begin{aligned} f(x) = \frac{L}{1+e^{-k(x-x_0)}} \end{aligned}$$

The reason for using these models is that they can capture many trends and patterns. In the first model, we adopt a pessimistic approach assuming that the exponential trend will continue indefinitely in the future. The second model captures many additives and multiplicative patterns in the data. Finally, the third model assumes convergence, which means that a stable state can be achieved.

### Point of Inflection and Limiting Value

In this study, we predicted the point of inflection and limiting value by using the generalized logistic function. The inflection point is the steepest part of the graph, representing the time of the most rapid growth of the curve. The limiting value is the population’s carrying capacity and shows us the total number of predicted cases in the final stage of the epidemic [23].

### Multivariate Time Series Models

When approaching time series forecasting, the most traditional statistical methods are autoregressive integrated moving average (ARIMA), exponential smoothing techniques [24], and vector autoregression (VAR) methods [25]. In machine learning, the most common technique to approach this problem is the long-short term memory (LSTM) network. However, other nonparametric algorithms can also be useful in this approach. This study compares the results of a traditional time series model (VAR) with a neural network model (LSTM) to better predict the number of cases and fatalities in Mexico.

### VAR

The vector autoregressive model is an extension of the univariate autoregression model for multivariate time series data. We decided to use this method, as VAR has proven to be one of the most suitable and flexible multivariate time series analysis models.

The model consists of a multi equation system that treats all variables as endogenous (dependent) and is a linear function of past observations [26]. The equation includes lagged values for each of the dependent variables in the system in its reduced form. This form is shown in equation 4 where $Y_t$ represents the vector of the time series variable, *a* is the vector of intercepts, $A_{i}$ is the coefficients matrices, and $\varepsilon _{t}$ is the vector of white noises.

4 $$\begin{aligned} Y_t = a + A_{1}Y_{t-1} + A_{2}Y_{t-2} + \cdots + A_{p}Y_{t-p} + \varepsilon _{t} \end{aligned}$$

### LSTM

The long-short term memory is an artificial recurrent neural network (RNN) architecture used in the field of deep learning [27]. This multilayered neural network can avoid the long-term dependency problem by adapting nonlinearities in the datasets [28], making it a significant performer in time series analysis [29].

This network’s concept consists of three nonlinear gates: the forget gate, the input gate and the output gate, and one “memory” cell. The “memory” cell transports relevant information through a sequence chain, and it can maintain its state value over a long time. In the process, the cell loses and wins information, and the gates are responsible for deciding what information should be added to the next time step and what should be removed. In these gates, the information is transformed with the logistic or sigmoid function into values between cero and one to make a “Yes”/“No” decision. A hyperbolic tangent($\tau$) is used to transform the information to values between -1 and 1 to make a ‘‘negative”/“neutral”/“positive” decision [28].

The role played by the first gate, the forget gate(f), is in deciding what is to be forgotten from the previous state data and which weighted previously hidden state information is to be remembered. The second gate, the input gate (i), determines what information is relevant to be written onto the Internal Cell State. Inside the LSTM cell unit, there are three outputs: C(t), y(t), and h(t); the calculation performed is shown in the equations below. Equations 5, 6, and 7, where $w_{0}$ represents the weights, $g_{0}$ represents a nonlinear function, which can be the sigmoid function, and *f f f*(*t*) represents an internal forget gate inside the input gate.

5 $$\begin{aligned} C(t) := f(t) C(t-1) + i(t) \end{aligned}$$

6 $$\begin{aligned} y(t) := g_{0}((w_{0} h(t))) \end{aligned}$$

7 $$\begin{aligned} h(t) := f \ f \ f(t) \tau (C(t)) \end{aligned}$$

Finally, the output gate(o9) determines what the output (hidden state) is from the Internal Cell State; this is achieved by multiplying the *f f f*(*t*) result by the current cell state with values from -1 and 1.

### Evaluation Metrics

We compute the following metrics to measure the performance of each of the models: The RMSE measures how close the fitted values are to the real values, and the Bayesian information criterion (BIC) to obtain the estimated likelihood to predict a model and to test how well the model fits the data [30]. The formulas for RMSE and BIC are shown in Eq. 8 and 9, respectively. In the RMSE formula, n represents the number of samples, $p_i$ is the forecasted values, and $o_i$ is the actual observed values. In the BIC equation, *K* is the number of model parameters, and the $\mathcal {L}$ is the maximized value of the likelihood of the model.

8 $$\begin{aligned} RMSE = \sqrt{\frac{1}{n}\sum _{i=1}^n (p_i - o_i)^2} \end{aligned}$$

9 $$\begin{aligned} BIC = - 2ln(\mathcal {L}) + K log(n) \end{aligned}$$

## Exploratory Data Analysis

**Fig. 1:**
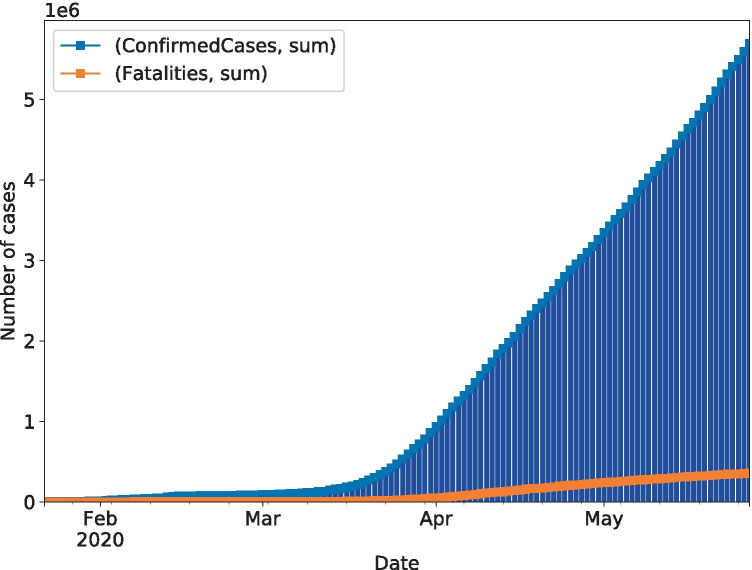
Accumulated worldwide COVID-19 confirmed cases since January 22, 2020

We compute the data’s main statistics during data exploration and perform analysis through graphs and plot visualization. First, we obtained an initial table from the data set variables (Table 1). A bar graph with the cumulative number of confirmed cases (blue line) and the number of fatalities (orange line) reported worldwide can be observed in Fig. 1.

**Table 1:** Statistical summary of confirmed cases and fatalities datasets

	Confirmed Cases	Fatalities
mean	5,757	380
std	49,991	3414
min	0	0
25%	0	0
50%	28	0
75%	547	0
max	1,699,176	100,417

Next, to visualize the pandemic’s course in Mexico compared to other Latin American countries, we evaluated the confirmed cases and fatalities from five different countries: Mexico, Chile, Brazil, Peru, and Ecuador. The statistics of each of the selected countries’ features are shown in Table 2. The growth factor of daily new infected cases is the daily new cases’ division by the total number of accumulated infected people in the previous day. The growth factor of daily new fatalities is the daily new fatalities’ division by the total number of accumulated fatalities. Finally, the average mortality rate is the number of daily fatalities divided by the daily cases.

**Table 2:** Data summary of five Latin America countries

Country	Mexico	Chile	Brazil	Peru	Ecuador
Start	2/28/20	3/3/20	2/26/20	3/6/20	3/1/20
End	5/21/20	5/21/20	5/21/20	5/21/20	5/21/20
Accumulated Mean Cases	16,153	16,337	77,318	32,060	13,775
St. Dev. of Accumulated	21668	20,796	110,300	42,013	14,005
Growth Factor of Daily New Cases	19.08%	17.24%	17.92%	19.93%	12.09%
Mean Fatalities of Daily Accumulated	2098	228	6,441	1,095	1,020
St. Dev Fatalities of Daily Accumulated	2471	221	7,503	1,191	1,102
Growth Factor of Daily New Fatalities	16.1%	12.84%	19.95%	12.49%	11.43%
Average Mortality Rate	5.67%	0.77%	4.18%	2.23%	4.3%

**Fig. 2:**
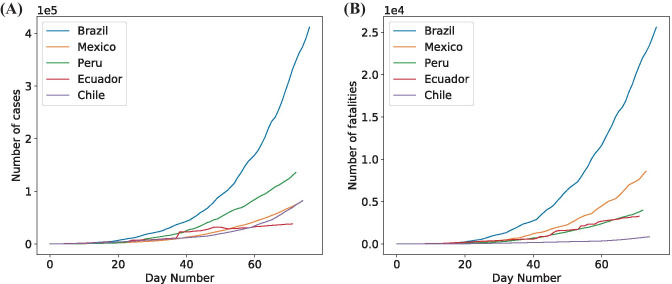
Total number of confirmed cases **(A)** and fatalities **(B)** since the confirmation of the first case

The countries of interest are plotted with their confirmed cases shown in Fig. 2a and fatalities shown in Fig. 2b. These graphs show the countries’ number of cases increasing over time. We can observe that even though Brazil has a higher number of daily cases, Mexico has a higher average mortality rate. We can also see some gaps in Ecuador’s data, as there are some sharp steps observed in the graph.

### Data Preparation

The data preparation phase consists mainly of data cleaning and feature reduction. The data set included confirmed cases and fatalities for Mexico, obtained from John Hopkins Repository, is clean in terms that it does not contain inconsistent or missing values. The same case is for climate data. Concerning the social mobility rate, there were missing values in the most recent dates. The data is until March 21st. Due to this limitation, the models used in this study consider this date as the last one.

**Fig. 3:**
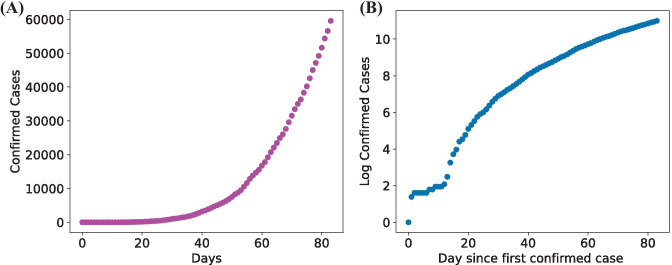
Accumulated number of confirmed cases **(A)** and logarithmic transformation of confirmed cases **(B)**

Besides, a data transformation was performed in the linear growth model’s dependent variable. As we identified in the visualization phase, the data does not have a linear evolution through time but exponentially. For this reason, we made a natural logarithmic transformation to the output variable (confirmed cases) to simulate a linear behavior and use it for prediction. Next, we included a row with the resulting logarithmic transformation in our table. Fig. 3b shows the log transformation. The data was transformed to add a new variable showing the number of days since the start date of the reported outbreak (January 22nd, 2019). The row shows day number 0 for January 22nd and day 69 for March 31st.

The time series models require additional preparation. It is essential to ensure the stationarity of all the time series variables before fitting the model. For this purpose, we first included a new column to transform the confirmed accumulated cases into daily cases by removing the number of infected people from the immediate prior date. Next, we performed logarithmic transformation on the daily number of cases and the daily number of fatalities. To transform the numeric values to a common scale, we used the z-score normalization method. And finally, we applied a smoothing technique.

Specifically, for the LSTM model, we must transform the time series data into a supervised learning problem. For this, we included the time lag variables for each of the covariates and dependent variables.

Next, to avoid overfitting problems and determine the most important input parameters, we performed feature selection using the Spearman Rank Correlation coefficient. This statistical method is used because it is robust when dealing with non-normally distributed data [31]. This method is a filer selection method, so we perform the selection before applying any machine learning algorithm.

Finally, to test each model’s performance, we separated the last 20% observations as a testing set and used 80% of the data to train the model. With this, we can use the hold-out data to test our predictions.

## Results

**Fig. 4:**
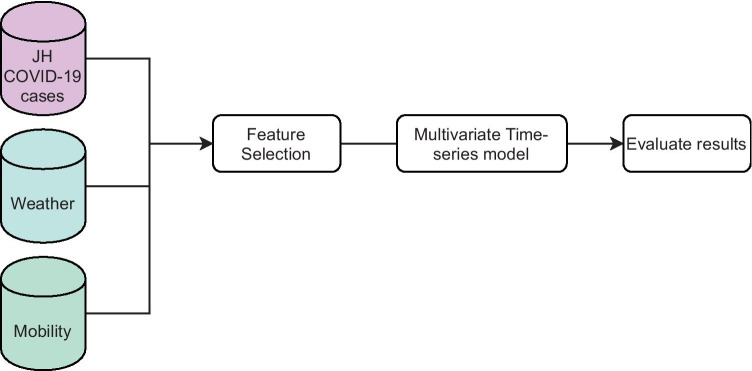
Constructed model diagram

This study evaluated the COVID-19-infected population growth in Mexico by comparing it with three curve fitting models: Linear, Polynomial, and Sigmoid Curve models, and then considered the generalized logistic growth model to determine the inflection point in Mexico. For this study’s second and third objectives, we used the Spearman Rank Correlation to select the most critical features and used these features in two time series models: VAR and LSTM. Finally, we compared the prediction results from these two models. The process followed in this second step is shown in Fig. 4.

### Population Growth Models

**Fig. 5:**
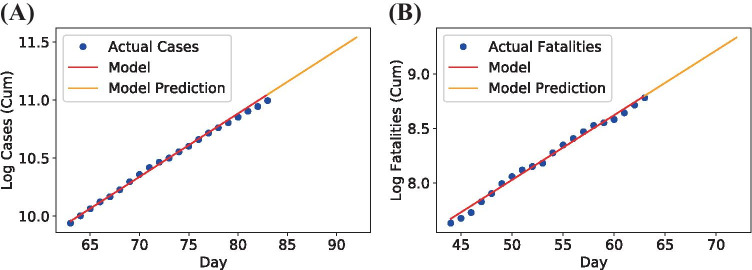
Linear regression model for logarithm confirmed cases **(A)** and fatalities **(B)** of Mexico of the last 20 days

We constructed the linear model with the last 20 days of data to understand if the growth behaved exponentially. The linear regression model for Mexico’s confirmed cases is shown in Fig. 5a and the accumulated number of fatalities in Fig. 5b. In the graph, we can observe that linear regression can accurately fit the logarithm data despite being a simple model. We can see that these last 20 days closely saw an exponential growth except for the latter observations.

The equation’s coefficients, Bayesian information criterion (BIC), and RMSE for both target variables are in Table 3. The RMSE is high for the confirmed cases in linear regression. It seems that the case numbers from Mexico are not behaving exponentially anymore. In contrast, Mexico Covid19 fatalities have a low RMSE, which indicates that the growth of deaths in the last 20 days fits this model well.

**Fig. 6:**
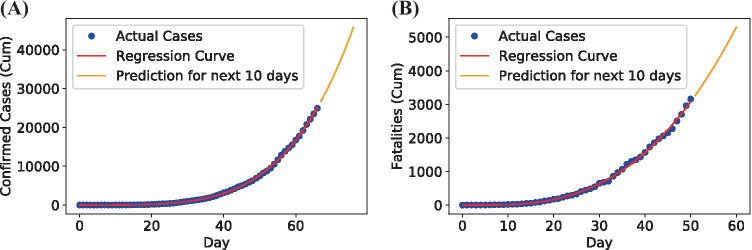
Polynomial regression model for Mexico confirmed cases **(A)** and fatalities **(B)**

The second model created to fit the data and predict coronavirus confirmed cases for the following weeks was a polynomial regression. Polynomial regression is a form of linear regression, where the dependent variable has an nth degree parameter. We performed a tuning process for this regression and obtained the best results with a fourth-degree parameter performed on 80% of the available data. The results obtained are shown in Table 3. The graphs in Fig. 6 show that the model fitted the data well only in the initial stage of infection.

**Table 3:** Growth models RMSE and BIC results with its corresponding coefficients

Models	Confirmed Cases			Fatalities		
	Coef.	RMSE	BIC	Coef.	RMSE	BIC
Linear Regression	c1=0.05 b=6.52	2299.24	80.62	c1=0.06 b=5.06	135.82	42.06
Polynomial Regression	c1=-16.67 c2=1.69 c3=-0.06 c4=0.01 b=34.14	3781.91	294.26	c1=-0.96 c2=-0.15 c3=0.03 c4=-0.01 b= 7.43	179.98	147.84
Sigmoid curve fitting	L=99,592 $x_0$=79.04 k=0.09	535.57	850.38	L=11,036 $x_0$=60.05 k=0.09	102.72	480.33

The third model used to fit the data and predict the confirmed cases of coronavirus for the following weeks is the GLM. The logistic function resembles a pandemic’s behavior, so the models created with this function are expected to follow this behavior. We implemented the curve fit function to get the best possible coefficients that adjust better to the training set’s data behavior. The results obtained for Mexico are shown in Table 3.

We can conclude from the results that the Sigmoid Curve, compared to the other two curve-fitting models, shows the best behavior of the infected population growth in Mexico for both the accumulated daily cases and daily fatalities.

**Fig. 7:**
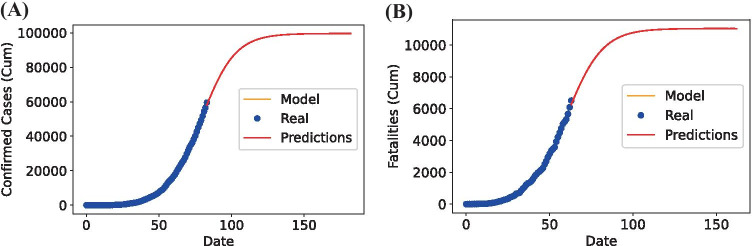
Sigmoid model for confirmed cases **(A)** and fatalities **(B)** of Mexico Sigmoid model for confirmed cases **(A)** and fatalities **(B)** of Mexico

Finally, this last model is used to make predictions for the next 100 days with the complete dataset’s input. We can see these predictions in Fig. 7. In the next section, the point of inflection and limiting parameters are obtained.

#### Point of Inflection and Limiting Value

This section predicts the point of inflection and limiting value by using the generalized logistic function. We determined the shape and some general features of the infection growth, and we can see that even with this simple equation and using only one variable, we can approach the curve behavior. Table 4 summarizes the results of both the accumulated cases and fatalities; these results consider that the lockdown’s compliance remains the same.

**Table 4:** Inflection point and limiting values of confirmed cases and deaths

	Accumulated Cases	Accumulated Fatalities
Inflection Point	49,796 (May 18th)	5,518 (May 19th)
Limiting value	99,592 (September 29th)	11,036 (August 27th)

### Feature Selection

**Fig. 8:**
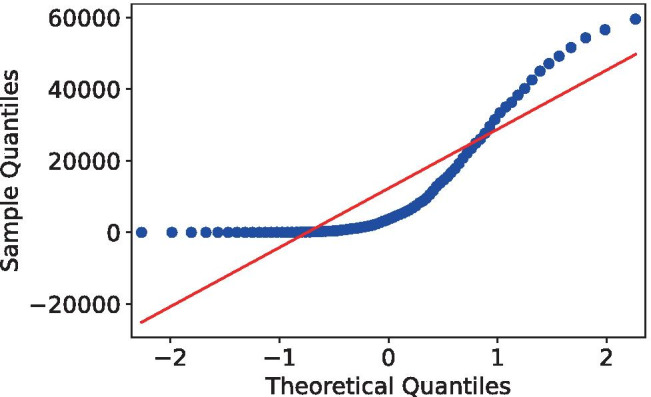
Non-normal distribution quantile–quantile scatter plot of confirmed cases

We performed a correlation analysis to determine the most significant input parameters, which will be used to build the multivariate time series models. To understand which correlation method to use for this, we first need to know if the data is normally distributed. To test normality, we used the Shapiro–Wilk test. The null hypothesis is that the data is normally distributed. The resulting statistic for the confirmed cases dataset was 0.76, with a p value smaller than 0.01. The fatalities dataset obtained a statistic of 0.82, with a p value smaller than 0.01. Based on these, we can conclude that there is enough statistical evidence to reject the null hypothesis. The plots in Fig. 8 show the data’s density behavior as a quantile-quantile plot for confirmed cases.

The following data were transformed into a supervised problem to obtain the t-n observations for the time series to understand which time lags and variables hold the highest linear coefficient correlation with the output variable. We used a Spearman coefficient matrix, as this is more robust when dealing with non-normality. Next, we calculated the absolute mean of each variable for each time step. The results are shown in Table 5.

**Table 5:** Time step new daily cases and fatalities feature selection coefficients

Variables	ID	New cases Absolute Mean	Fatalities Absolute Mean
New log cases	var1	0.84	0.53
Max tempC	var2	0.30	0.26
Min tempC	var3	0.31	0.24
UV Index	var4	0.23	0.19
Humidity	var5	0.30	0.21
PrecipMM	var6	0.33	0.26
Pressure	var7	0.24	0.19
Wind speed Kmph	var8	0.15	0.15
Retail and Recreation	var9	0.32	0.34
Grocery and Pharmacy	var10	0.38	0.32
Parks	var11	0.35	0.35
Transit stations	var12	0.40	0.35
Workplaces	var13	0.21	0.35
Residential	var14	0.21	0.33

We identified that the top five features with the highest correlation with respect to the daily cases are the amount of cases at $t-14$ (0.92) , the transit stations mobility rate $t-28$ (-0.79), parks mobility rate at $t-26$ (-0.76), grocery and pharmacy mobility rate at $t-26$ (-0.75), and maximum temperature ($^{\circ }$C) at $t-28$ (-0.70). In contrast, for the daily fatalities the top five features with the highest correlation with respect to the daily fatalities are the daily cases at $t-7$ (0.84), the residential mobility at $t-28$ (0.78), parks mobility rate at $t-1$ (0.75), transit stations at $t-28$ (-0-.71), and grocery and pharmacy mobility rate at $t-25$ (-0.70).

The defined threshold used for the confirmed cases and fatalities was 0.30 and 0.25, respectively. All attributes that had an absolute mean value smaller than 0.30 were eliminated (UV index, pressure, wind speed, workplaces mobility rate, and residential mobility rate) from the confirmed cases data frame. Concerning fatalities, all attributes with an absolute mean Spearman correlation coefficient value less than 0.25 were eliminated (minimum temperature, UV Index, humidity, pressure, and wind speed).

### Multivariate Time Series Models

In this phase, we compare the scores of two multivariate time series models (LSTM and VAR) to identify which one is the best at predicting the new daily cases and fatalities caused by COVID-19 in Mexico.

As autoregressive models perform better when the time set is stationary, we performed an Augmented Dickey-Fuller (ADF) test to prove stationarity. The null hypothesis of this test is that the data set has a unit root and is nonstationary. The resulting statistic was -1.43 for confirmed cases and 0.52 for fatalities. As the resulting p value (0.58, 0.99) of confirmed cases data and fatalities, respectively, were higher than the defined significance level of 0.05, we do not have sufficient statistical evidence to reject the null hypothesis with a 95% confidence level.

Therefore, we transformed the data into a logarithm scale, and a new output variable was created by using the differencing method. After the transformation, we shrunk the time series to 56 preview days. We again performed the ADF test on the transformed data. The resulting statistic was -3.06, with a p value of 0.03 for the confirmed cases dataset. Regarding the fatalities dataset, the resulting statistic was -4.12 with a p-value of $8.81\mathrm {e}{-4}$. We have sufficient statistical evidence to reject the null hypothesis. The model can improve its forecast capability by applying a smoothing technique. For this analysis, we used the single exponential smoothing with a smoothing constant of 0.2. We decided on the value that maximizes the MSE after experimenting with different constant values and evaluating the model with new information never seen by the model. Finally, after smoothing the data, we normalized it with the z-score function.

Following this preparation, we fitted the VAR and LSTM models. After a series of experiments, the VAR model showed the best results with a lag order of seven for both data frames. To select the lag order, we fitted the model with different values with the BIC metric as the evaluator. Due to the number of coefficients in the resulting VAR model, they are not included in this paper but are available on request.

For the neural network model, we used a two-layer LTSM with 200 neurons in the first layer, 100 in the second layer, and a time lag of 28 days. The results are shown in Table 7. For this analysis we used $t_{0}$ as of May 21st. The hyperparameters are shown in Table 6.

**Table 6:** Hyperparameters of LSTM

Hidden Layers	2
Number of neurons in hidden layer 1	200
Activation of hidden layer 1	tanh
Number of neurons in hidden layer 2	100
Activation of hidden layer 2	tanh
Batch size	100
Epochs	100
Loss function	MSE
Optimizer	Adam

The results of the models are shown in Table 7. The computed RMSE and AIC help us to compare the resulting models to select the one with better performance. The values indicate that the best model for predicting daily cases and daily fatalities’ is the LSTM model with an RSME smaller in 47.16% for the confirmed cases and 33.27% for the fatalities dataset.

**Table 7:** Time series models metrics summary

Model	RMSE	BIC
LSTM daily cases	275.35	71.00
LSTM daily fatalities	31.91	45.14
VAR daily cases	630.3469	94.14
VAR daily fatalities	208.4456	78.65

## Discussions

In the present study, we trained an LSTM network with data from January 22 to March 22, 2020, as reported by the Mexican government and provided to John Hopkins University of Medicine. Firstly, we have shown how the daily cases and fatalities predictions are greatly affected by the quarantine hence the decisions made by the government to decrease the spread are crucial. We have also proven the importance of integrating weather variables to respiratory viruses’ prediction models, especially the maximum daily temperature. Finally, we have demonstrated that the predictions obtained from a recurrent neural network can yield better performance than a traditional time series model. The RMSE result of our initial LSTM model was 47.16% smaller than our VAR model for confirmed cases and 33.27% smaller for fatalities. Months after the creation of our initial model, we have validated the model with updated data. We have provided data from October 14 to November 3, 2020, and the resulting RMSE of the LSTM obtained was 1410.57 for confirmed cases and 264.42 for deaths.

There are several challenges and limitations considered in the modeling of the COVID-19 cases. In summary, this study’s limitations include the data collection bias, the number of reported cases that are in function of the number of tests that are applied, and the government’s willingness to report the numbers. A potential censoring in the data can affect the predictions. Therefore, the model’s training depends on the number of daily cases registered based on the number of available tests that undoubtedly exist. Thus, there are zero known cases in the data when there are zero tests, but this does not necessarily reflect the reality; in other cases, there are observations with a spike in daily confirmed cases, indicating that there were many tests available. One last identified limitation is the lack of sufficient data, which may deter the predictions. We consider these limitations as present in the reported cases of Mexico. However, we firmly believe that forecasting the number of daily cases and deaths is essential and required to support the government’s health care institutions and the decision-making process.

In this investigation, we have compared a logarithmic curve fitting model with a linear and polynomial fit and confirmed that the sigmoid function is the best approximation of the disease’s behavior for five Latin American countries. These results are in consent to the study from Schuttler et al. [12], which showed this behavior for different European countries and from the analysis performed by Andreas et al. [13], which among other discoveries estimated the inflection points for Italy with a 0.99 result in the coefficient of determination.

Additionally, in this study, we have confirmed the importance of including weather factors to predict daily cases. These factors have also been observed by Liu [8] who explored their influence in predicting COVID transmissions in China. Compared to Chakraborty and Ghosh [9], who proved that ARIMA models showed better results in Canada and UK, we have shown how neural networks can achieve better results than traditional time series forecasting to predict cases in Mexico. Moreover, this study, just like the work of Tomar and Gupta [10], and Chimmula and Zhang [11], stress the importance of social isolation. Considering that the social distancing measures were conserved, our model predicted that the maximum value of new cases in Mexico would be reached in September 2020.

We can use several approaches to extend this study. For instance, the application of generative learning models, such as Hidden Markov Models, could serve useful for our prediction since it has proven to be efficient when having few data points [9, 32]. Given that accumulating studies have recognized respiratory virus as generally seasonable [33–35], we could improve our model by supplying it with the weather, mobility, and infection data for one full year. Finally, we can update the model created in this study with the latest information to assist healthcare experts and policymakers. This model can predict the number of cases and fatalities for other countries since it has already proven useful in the multiple countries analyzed in this paper.

## Conclusion

The results of the curve fitting model estimated the inflection point on May 15, 2020. With this, we predicted that the maximum limit value of the outbreak in Mexico would be reached around the end of September, with the prediction limitation that the lockdown remained in place. In this study, we also identified several relational features to predict COVID-19 daily cases. We detected that the features with the highest correlation to the daily cases and fatalities were the following: the number of cases, the transportation station mobility, park mobility, the grocery pharmacy mobility, the residential mobility, and the maximum daily temperature ($^{\circ }$C). Finally, we demonstrated that it is better to use an LSTM network for this prediction instead of the traditional statistical model of VAR, as we obtained better results with an RSME smaller in 47.16% for the new cases and 33.27% for fatalities.

With this study, we contribute to the literature by applying deep learning techniques to identify the pandemic impacts in Mexico and compare them to more traditional and powerful forecasting methods, VAR, and statistical curve-fitting methods. We hope that this study contributes to the world’s response to the SARS-CoV-2 epidemic, applying machine learning for complementing the state-of-the-art mathematical models and providing some references for future research.

## Footnotes

1 https://github.com/DRAE-1 MLP/COVID19_Mexico
